# PSMA Expression in Solid Tumors beyond the Prostate Gland: Ready for Theranostic Applications?

**DOI:** 10.3390/jcm11216590

**Published:** 2022-11-07

**Authors:** Chiara Lauri, Lorenzo Chiurchioni, Vincenzo Marcello Russo, Luca Zannini, Alberto Signore

**Affiliations:** Nuclear Medicine Unit, Department of Medical-Surgical Sciences and of Translational Medicine, “Sapienza” University, 00161 Rome, Italy

**Keywords:** PSMA, cancer, theranostics, PET/CT, radioligand therapy

## Abstract

In the past decades, the expanding use of prostate-specific membrane antigen (PSMA) imaging for prostate cancer has led to the incidental detection of a lot of extra-prostatic malignancies showing an increased uptake of PSMA. Due to these incidental findings, the increasing amount of immunohistochemistry studies and the deeper knowledge of the mechanisms of expression of this antigen, it is now clear that “PSMA” is a misnomer, since it is not specific to the prostate gland. Nevertheless, this lack of specificity could represent an interesting opportunity to bring new insights on the biology of PSMA and its sites of expression to image and treat new conditions, particularly several cancers. In this review, we will describe the main extra-prostatic cancers that exhibit PSMA expression and that can be studied with PSMA-based positron emission tomography–computed tomography (PET/CT) as an additional or alternative tool to conventional imaging. In particular, we will focus on cancers in which a radioligand therapy with ^177^lutetium has been attempted, aiming to provide an overview of the possible future theragnostic applications of PSMA.

## 1. Introduction

In the last decade, the scientific community has shown a considerable and growing interest in prostate-specific membrane antigen (PSMA) due to its favorable characteristics of expression, which make it a very attractive target for molecular imaging agents. PSMA is a transmembrane type II glycoprotein, encoded by the FOLH1 gene, composed of intracellular, transmembrane and extracellular domains with catalytic activity. After binding to the extracellular domain, the ligand is internalized and undergoes endosomal recycling, resulting in increased deposition, tumor uptake and retention and offering encouraging prospects for both diagnostic and therapeutic purposes [1].

The discovery of PSMA, at the beginning of the 1980s, was strictly related to the prostate gland, where it is normally expressed. Higher levels of PSMA of up to 1000-fold have been detected in secretory cells of prostate cancer (PCa) in the epithelium, and from several studies it emerged that there is a correlation with high Gleason scores, high serum levels of prostate specific antigen (PSA), advanced stages of disease and metastatic castration-resistant PC (mCRPC) [2,3,4]. Even if PSMA’s functions are not completely understood yet, it contributes to tumor invasion via different molecular pathways and it plays a role in matrix degradation and the regulation of angiogenesis [1]. For these aspects, PSMA represents an attractive target for labelling with both diagnostic and therapeutic isotopes.

Currently, the most used molecular imaging agents for positron emission tomography–computed tomography (PET/CT) include gallium-68 (^68^Ga)-labelled PSMA ligands, such as ^68^Ga-PSMA-11 (also known as ^68^Ga-PSMA, ^68^Ga-PSMA-611, ^68^Ga-PSMA-HBED-CC and ^68^Ga-DKFZ-PSMA-11), and the fluorinated agents ^18^F-DCFPyL and ^18^F-PSMA-1007 [5,6]. ^18^F-labelled compounds have the known advantages of lower positron energy and longer half-life values compared to ^68^Ga; therefore, it is possible to perform a higher number of PET/CT examinations and to distribute the isotopes in distant areas [7]. Nevertheless, the major disadvantages of ^18^F include the need for cyclotron, which is more expensive compared to the ^68^Ga generator, and the more complex and time-consuming labelling procedure. Therefore, due to the wide availability of ^68^germanium (^68^Ge)/^68^Ga generators in many nuclear medicine (NM) departments, ^68^Ga-labelled PSMA is nowadays largely used worldwide.

**Figure 1:**
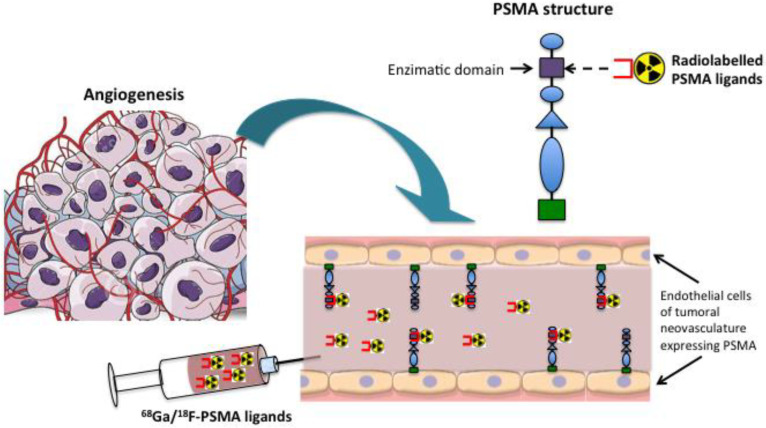
Mechanism of expression of PSMA in the neovasculature of non-prostatic tumors.

In the last decades, the PET imaging of PCa has gradually shifted from choline to PSMA, due to its higher sensitivity, even in patients with low PSA values, allowing the prompt identification of local or distant recurrences before they become positive for ^18^F-choline. With the expanding adoption of PSMA-based imaging agents, a lot of extra-prostatic tissues showing an increased uptake of PSMA have been detected, bringing new insights to PSMA’s biology and sites of expression. Due to these incidental findings, the increasing amount of immunohistochemistry (IHC) studies and the deeper knowledge of the mechanisms of expression of this antigen, it is now clear for the scientific community that the name “PSMA” is not appropriate. It has, indeed, been identified in several other normal tissues (such as renal tubules, salivary and lacrimal glands, the small and large bowels, astrocytes, the liver, the spleen, the thyroid gland and synovial tissue), in many non-neoplastic conditions (infective or inflammatory processes, bone-related conditions, benign diseases) and in a lot of non-prostatic malignancies. Interestingly, in extra-prostatic tumors, PSMA is mainly expressed on the endothelium of the neovasculature rather than the epithelium [8] (Figure 1). This would suggest new future applications for PSMA radioligand therapy (PRLT).

This review provides an overview of a wide spectrum of non-prostatic diseases expressing PSMA, and in particular it will focus on tumors in which a PRLT therapy with lutetium-177 (^177^Lu)-PSMA has been attempted (Table 1), summarizing the possible theranostic properties and future directions of this agent.

## 2. Salivary Glands Cancer

Salivary glands represent a site of physiologic uptake of ^18^F or ^68^Ga-PSMA, representing one of the major concerns when planning a PRLT due to the possible radiation-induced damage. Several studies report increased uptake, not only in healthy salivary glands, but also different histotypes of salivary gland tumors, such as adenocistic carcinoma (AdCC) and salivary duct carcinoma (SDC), raising the problem of how to differentiate a physiologic uptake from a pathologic finding. However, the mechanism of uptake of PSMA radioligands in salivary glands is still controversial and seems to be non-specific and non-completely mediated by PSMA [9].

Nishida et al. performed IHC when studying PSMA expression in 55 salivary gland cancers, including pleomorphic adenomas, Warthin tumors, basal cell adenomas, AdCC, mucoepidermoid carcinomas and salivary duct carcinomas (SDC). PSMA expression was detected in 97% of benign tumors, in 77% of malignant tumors and in 59% of normal salivary glands close to the tumor. In most cases, PSMA expression was detected in both luminal and myoepithelial cells; however, in pleomorphic adenomas and basal cell carcinoma, the myoepithelial cells showed significantly stronger staining than the luminal cells. The expression of PSMA also in normal glands deserves the adoption of special caution when planning a PRLT [10]. Interestingly, from other IHC studies, it emerged that different salivary gland tumors show very heterogeneous behaviour of the expression of PSMA. Indeed, no adrenocortical carcinomas express PSMA in the neovasculature, whereas in SDCs PSMA is expressed by both the neovasculature and by tumor cells [11,12,13].

Van Boxel et al. described ^68^Ga-PSMA uptake in patients with AdCC and SDC, and the maximum standardized uptake values (SUVmax) in tumor lesions were correlated with PSMA expression via IHC. In AdCC patients, the SUVmax values ranged from 1.1 to 30.2, with a tumor/liver ratio >1 in 93% of patients. In patients with SDC, the SUVmax values ranged from 0.3 to 25.9, with a tumor/liver ratio >1 in 40% of patients. They found a large intra-patient inter-metastatic variation in uptake of ^68^Ga-PSMA in both histotypes, which can have an influence on the treatment response. Moreover, they found a negative correlation between the intensity of uptake via PET/CT and the time from diagnosis in SDC patients, suggesting the planning of a PRLT in early phases of diseases, differing from PCa, in which PRLT is generally reserved to the advanced stages of diseases. Interestingly, the use of IHC did not predict ligand uptake in AdCC and SDC patients [11].

Isgoren et al. studied 2 cases of metastatic AdCC with both ^18^F-fluoro-deoxyglucose (FDG) and ^68^Ga-PSMA. The first patient studied for tongue AdCC staging showed pathological FDG and PSMA uptake either in the primary tumor (SUVmax 12.8 with FDG and SUVmax 7.1 with PSMA) or in the metastatic neck lymph nodes (with similar uptake intensity levels). The second patient, after the surgical removal of an AdCC in the nasal cavity, was studied with PET/CT scans for the evaluation of new liver metastases detected by magnetic resonance imaging (MRI). The ^18^F-FDG PET/CT results showed the presence of two bone lesions and two metastatic liver lesions. The ^68^Ga-PSMA PET/CT images showed multiple PSMA-avid metastatic bone and liver lesions. Most of these lesions were not detected via FDG-PET, suggesting that PSMA may be more sensitive than FDG in salivary gland malignancies and metastases. However, larger prospective studies are needed to assess the possible value of PSMA-based imaging as an alternative—or in addition—to ^18^F-FDG for salivary gland tumor staging and restaging [14].

The increasing evidence of cancer localizations on salivary glands with PSMA- PET/CT, encouraged some authors to explore the possible use of a palliative PSMA target therapy with ^177^Lu-PSMA in these kind of patients after the failure of conventional treatments (Table 1). Klein-Nulent et al. published the first report on 6 patients with recurrent or metastatic salivary gland cancer (4 cases with AdCC, 1 case with salivary gland adenocarcinoma and 1 with acinar cell carcinoma) who had no other standard palliative care options. The patient tumor PSMA status was assessed using an IHC analysis of PSMA expression in tumor tissues and via the evaluation of PSMA ligand uptake levels using ^68^Ga-PSMA-PET/CT. The treatment plan consisted of 4 cycles of 6.0–7.4 GBq of ^177^Lu-PSMA-617 administered intravenously every 6–8 weeks. In four out of the six patients, therapy was discontinued, because of disease progression after two cycles (in 2 patients) and due to grade 1 side-effects, mainly fatigue and xerostomia, nausea and bone pain (in 1 patient) and the development of a severe grade 3 thrombocytopenia (in 1 patient). Nevertheless, no treatment-related death was reported. Interestingly, a radiologic response, showing stable disease or partial response, was observed in two patients, and four patients reported the immediate relief of tumor-related symptoms. The authors concluded that palliative PSMA-targeted RLT therapy for advanced or metastatic salivary gland cancer with ^177^Lu-PSMA may be effective, is relatively well tolerated and it could significantly relieve tumor-related symptoms when tumor targeting is sufficient. Nevertheless, larger studies are needed to confirm these conclusions [15].

## 3. Thyroid Cancer

**Figure 2:**
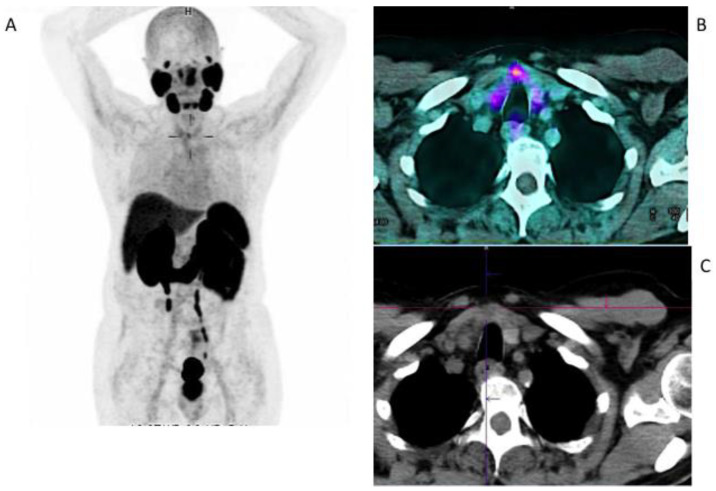
Incidental findings of thyroid cancer in a 63-year-old patient studied with ^68^Ga-PSMA-11 for prostate cancer. MIP (**A**) and fused images (**B**) showing focal uptake (SUVmax 3.5) on the thyroid gland corresponding to a hypo-dense nodule on a co-registered low-dose CT scan (**C**). The ultrasound fine needle aspiration revealed Thy 4 (suspicious for malignancy) and the patient underwent a total thyroidectomy. The histology revealed a poorly differentiated papillary thyroid carcinoma.

The global incidence of thyroid cancer has increased in the past decades due to the improved accuracy of diagnostic methodologies, the higher awareness of the population to screening programs and increased incidental detection in imaging examinations or laboratory tests [16]. While differentiated thyroid cancer (DTC) has an excellent overall survival (OS) rate if properly treated, poorly differentiated thyroid carcinoma (PDTC) and anaplastic thyroid cancer (ATC) show poor prognoses due to their aggressiveness and metastatic spread at diagnosis. However, even DTC may show a progressive de-differentiation during the course of the diseases, becoming radioiodine-refractory and precluding the possibility to non-invasively treat the patients with 131-iodione (^131^I) [17]. After several incidental findings of increased ^68^Ga-PSMA uptake in the thyroid gland in patients with PCa who underwent PSMA PET/CT imaging (Figure 2), the histological PSMA expression in thyroid cancer was evaluated.

According to the systematic review performed by Bertagna and colleagues, a malignant tumor was diagnosed in up to 23% of patients showing increased PSMA uptake in the thyroid gland [18]. Bychkov et al. studied a large variety of thyroid specimens with IHC, showing that the PSMA expression is attributed to endothelial microvessels of thyroid cancer, and just in one case of PDCT on the surfaces of follicular cancer cells. Conversely, non-neoplastic parenchyma or non-neoplastic vessels did not exhibit PSMA expression [19]. In particular, they found that the PSMA expression in the endothelium of the tumor-associated vasculature is notably higher in PDTC, ATC and iodine-refractory thyroid cancers, concluding that this feature can be considered a biomarker of aggressiveness, in accordance with its important role in tumorigenesis and angiogenesis. Wachter et al. investigated in detail the PSMA expression levels in 39 patients with ATC and 22 with PDTC via an IHC analysis of both primary tumors and metastases, concluding that 36/39 ATCs were PSMA+ (high intensity in 27 patients) and 21/22 PDTC were PSMA+ (high in 13 patients). Moreover, from the comparative analyses, it emerged that the PSMA expression was higher in the primary tumors than metastatic sites due to their different microenvironments and signalling pathways. Furthermore, 7 out of 8 patients who underwent both ^18^F-FDG PET/CT and ^68^GaPSMA PET/CT showed higher FDG uptake rates compared to PSMA uptake, showing that ^18^F-FDG-PET/CT still has higher accuracy than ^68^Ga-PSMA PET/CT in detecting more aggressive thyroid cancer histotypes [20]. On the other hand, de Vries et al. retrospectively analyzed a small cohort of 5 patients affected by radioiodine-refractory thyroid cancer by using the dual-tracer approach (^18^F-FDG and ^68^Ga-PSMA PET/CT). In this small series, ^68^Ga-PSMA allowed the detection of three additional lesions in two patients that were negative at ^18^F-FDG PET/CT, concluding that PSMA PET/CT can be useful in cases of FDG-negative findings or for patients that are non-responsive to conventional treatments [21]. Another group achieved similar conclusions by prospectively investigating 11 patients affected by metastatic thyroid cancer with ^18^F-FDG PET/CT and ^68^Ga-PSMA PET/MRI and reporting a great heterogeneity in FDG and PSMA uptake rates within cancer subtypes and even within individual patients. The detection rate of FDG PET/CT (41/43 metastases) was higher than in PSMA PET/MRI (29/43 metastases), but the only two FDG negative lesions (both osseous) were PSMA-positive. Moreover, different patients with the same histological subtypes showed different results in the FDG and PSMA scans, showing that the specific subtype alone is not sufficient to predict PSMA uptake [22].

Several studies also investigated the potential utility of a PSMA PET/CT scan in DTC. Verma et al. compared ^131^I whole-body scan (WBS), ^18^F-FDG and ^68^Ga-PSMA PET/CT results in 10 metastatic DTC patients with 32 lesions; all patients with iodine-avid disease showed PSMA uptake in the corresponding lesions, with the only exception being 2 local recurrences in the thyroid bed, which were PSMA-negative and iodine-positive. PSMA was detected in 30/32 total lesions, while FDG was detected in 23/32 lesions [23]. In a recent retrospective study, Pitalua-Cortes et al. compared post-^131^I treatment WBS with SPECT/CT and ^68^Ga-PSMA PET/CT results in 10 patients with advanced DTC; 64/64 lesions were detected via PSMA PET/CT, while only 55/64 were detected via ^131^I SPECT/CT, showing that the PSMA expression could be used as a predictive biomarker of radioiodine-refractoriness [24]. Indeed, the prompt identification of patients who are showing a progressive de-differentiation of thyroid cancer could be extremely important considering that it is associated with poorer clinical outcomes and with 5-year disease-specific survival rates of 60% to 70%, and considering that its therapeutic management can be a challenge for the clinicians [25].

As far as the possible application of PRLT in thyroid cancer is concerned, few data are available at the moment and no clear indications have been proposed to determine the eligibility for PSMA RLT. Moreover, applying the same criteria commonly used for PCa (for example, SUVmax values in dominant sites on ^68^Ga-PSMA PET/CT at least 1.5 times those of the SUVmean values in the liver) could be misleading, considering the different PSMA mechanisms of expression in non-prostatic tumors (namely in the endothelium of tumor microvessels) [26].

To the best of our knowledge, only a few patients treated with ^177^Lu-PSMA RLT are reported in literature (Table 1). Assadi et al. reported a metastatic radioiodine-refractory DTC patient who underwent, in the following order, ^131^I therapy, sorafenib, 1 cycle of ^177^Lu-DOTATATE (7.4 GBq) and finally for disease progression 1 cycle of ^177^Lu-PSMA RLT (7.4 GBq). The WBS after treatment showed higher uptake of PSMA than the somatostatin analogue in the metastatic sites. Unfortunately, the response assessment for ^177^Lu-PSMA RLT was not possible because the patient died two weeks later from a cardiac attack [27]. Two of the five patients evaluated by de Vries et al., as mentioned above, also underwent 2 cycles of ^177^Lu-PSMA RLT [21]. One of these patients showed progressive disease after one cycle and the second showed a transient (7 months) decrease in tireoglobulin (Tg) levels, increased levels of anti-Tg antibodies and a partial response upon imaging. In particular, the ^68^Ga-PSMA PET/CT performed 5 weeks after the second cycle showed size decreases in several lung lesions, response of the liver metastases and stable disease of the cervical metastases [21]. Finally, Wächter et al. reported one PDTC patient who received two cycles of ^177^Lu-PSMA therapy, based on high PSMA uptake based on PSMA PET/CT and ^18^F-FDG-negative scans, with a time interval of 8 weeks (cumulative dose 13.7 GBq), showing stable disease for 7 months, followed by an increase in Tg levels and the appearance of ^18^F-FDG-positive metastases [20].

In summary, PSMA PET/CT seems to perform better in DTC, especially in cases of radioiodine-refractoriness, rather than PDTC and ATC, for which ^18^F-FDG PET/CT has higher diagnostic accuracy. Some selected patients may benefit from the dual-tracer approach, but more and larger prospective studies are needed to assess the role of PSMA PET/CT in the disease monitoring of these patients and to better explore the performance of the ^177^Lu-PSMA RLT in selected thyroid cancers.

## 4. Hepatocellular Carcinoma

Hepatocellular carcinoma (HCC) is the most frequent primary liver neoplasm and is the fourth most common cause of cancer death worldwide [28]. Diagnosis is generally achieved by performing radiologic examinations, such as ultrasounds (US), contrast-enhanced CT, MRI and histology testing. NM plays only a marginal role; rather, it is more useful in detecting liver metastases from other tumors. ^18^F-FDG PET/CT indeed has a low sensitivity for HCC, especially in well-differentiated or moderately differentiated histotypes, which usually show a weak lesion uptake that is comparable to those observed in normal parenchyma. This is maybe due to the increased glucose-6-phosphatase activity in HCC cells [29]. Conversely, intense ^18^F-FDG uptake is observed in poorly differentiated lesions, being associated with a worse prognosis [30]. Recently, with the increasing number of case reports of the incidental uptake of PSMA in HCC lesions and the IHC confirmation of PSMA expression in the neovasculature of HCC [8,31,32,33], some authors speculated whether PSMA-targeted PET/CT could be useful to evaluate the expression of this antigen by the tumor and to select patients who would benefit from a tailored PRLT after the failure of conventional treatments. Gündoğan and colleagues prospectively studied 14 patients with HCC who underwent MRI, ^18^F-FDG PET/CT and ^68^Ga-PSMA PET/CT [34]. The MRI and ^68^Ga-PSMA PET/CT were able to detect a significantly higher number of liver lesions compared to ^18^F-FDG. Moreover, most of the patients showing low or moderate ^18^F-FDG uptake showed high PSMA uptake. Conversely, in one patient the PSMA uptake was weak and the ^18^F-FDG uptake was high. Additionally, the detection rate of abdominal lymph nodes was higher for PSMA compared to ^18^F-FDG, and interestingly mediastinal lymph nodes showing high metabolic activity via ^18^F-FDG in one patient were negative for PSMA. The histopathological examination performed in this patient showed the presence of anthracosis, supporting the idea that PSMA-based imaging could offer more accurate staging in patients with HCC. Moreover, in this series, histology testing was performed in 6 out of 14 patient and the grades of de-differentiation were correlated with different uptake rates for both radiopharmaceuticals, with poorly differentiated HCC being characterized by similar uptake rates for both isotopes, and with well-differentiated HCC showing low ^18^F-FGD uptake and high PSMA uptake. Nevertheless, due to the small sample size in this study and the lack of other comparative studies using histology testing as the gold standard, a definite conclusion cannot be reached. Other groups also applied a dual-tracer PET approach to HCC patients, reporting similar findings and suggesting that the use of PSMA-based imaging could offer a valuable tool in T and N staging in addition to MRI, opening new perspectives for planning a PRLT [32,33]. This aspect also emerged in the study by Hirmas et al., who retrospectively compared the accuracy rates of ^68^Ga-PSMA-11 PET/CT and CT in detecting HCC lesions. They found comparable detection rates for both modalities for staging liver lesions, with higher performance of ^68^Ga-PSMA PET/CT in the detection of extra-hepatic lesions [35]. The use of PET/CT allowed accurate upstaging and downstaging of the patients, allowing changes to the management approach in a considerable percentage of patients. Nevertheless, despite the encouraging results derived from PSMA imaging, a PRLT was attempted only in 2 patients, with very poor results [35] (Table 1). They both underwent one cycle of ^177^Lu-PSMA RLT (activity 5.9–6.2 GBq), but the SPECT/CT-based dosimetry approach showed at least a ten-fold lower radiation dose than that achieved with one cycle of external beam radiation; therefore, the treatment was discontinued in both patients, meaning it was not superior to conventional approaches [35].

## 5. Renal Cell Carcinoma

Renal cell carcinoma (RCC) represents more than 90% of kidney cancer cases, counting for 3 to 5% of cancer diagnoses, with a slightly higher prevalence in males [36]. The most common histotypes include clear renal cell carcinoma (CRCC), papillary renal cell carcinoma (PRCC) and chromophobe renal cell carcinoma (ChRCC) [37].

From IHC studies, it is now clear that PSMA is physiologically expressed by the endothelium in the proximal renal tubules of normal kidneys. During the malignant transformation, the expression can be observed in newly formed vessels, particularly in the clear cell histotype (84%), in which the pro-angiogenic factors (PDGF, VEGF) are strongly upregulated, and to a lesser extent in ChRCC (61%) and PRCC (28%) [38]. Spatz et al. evaluated the PSMA expression in patients affected by CRCC, ChRCC and PRCC, showing that in all histotypes only the endothelia of the tumoral microvessels were PSMA+ under IHC, with the exception of two PRCC patients, in which cytoplasmatic PSMA expression was observed in the specimens. By correlating PSMA expression with the overall survival (OS), the authors found that a high PSMA staining intensity was associated with poorer OS [39].

The expression of PSMA in both healthy kidneys and in the tumor-associated neovasculature of primary and metastatic tissues could make the use of PSMA-targeted imaging very useful in the staging and restaging of RCC. This approach would find an application in particular for the evaluation of distant metastases, rather than local disease, due to the low T/B ratio [38,40,41,42]. PSMA PET/CT imaging, with both ^68^Ga and ^18^F, has been attempted by several authors, mainly in CRCC, demonstrating promising results in the detection of bone, lymph node, soft tissue and abdominal metastases. However, the available literature is scarce and limited to case reports or studies with small sample sizes [40,41,43,44,45,46,47,48,49,50]. Sasikumar et al. described a patient with metastatic CRCC studied with both ^18^F-FDG and ^68^Ga-PSMA, reporting significantly higher uptake of mediastinal lymph nodes, suprarenal region, bone metastases and thyroid nodules with ^68^Ga-PSMA compared to standard ^18^F-FDG [44]. Other comparative studies were performed by other groups [45,46,47,48,49] on a total of 12 patients. In all of these studies, PSMA PET/CT detected more lesions than ^18^F-FDG, supporting the possible utility of this approach as an alternative to ^18^F-FDG, which has many limitations in clinical practice for studying renal cancers. PSMA PET/CT could indeed be more sensitive than^18^F-FDG, because from these preliminary results, changes in PSMA expression and uptake precede the phase in which the tumors become FDG-avid. Therefore, the introduction of PSMA PET/CT in the early diagnostic workup of such neoplasms could be very useful for staging and therapy planning in patients with oligo-metastatic disease. Indeed, several groups investigated the role of PSMA-PET imaging for the initial staging of CRCC, reporting its usefulness in the assessment of distant metastases, which showed unclear findings for conventional imaging [41,43,45,46]. Nevertheless, Yin and colleagues did not achieve good results in non-clear cell renal carcinomas, highlighting that different histotypes may show different biological behaviors that influence PSMA expression [50]. Finally, PSMA expression in these types of tumors may open, in the future, the possibility to treat these patients with ^177^Lu-PSMA, possibly revolutionizing the classical therapeutic strategies [47]. Nevertheless, at the moment no patients affected by renal cancer have been treated with PRLT.

## 6. Glioblastoma

Glioblastoma, a tumor derived from glia cells, is the most common brain cancer, with an annual incidence rate of 5/100,000 people [51]. It is a highly vascularized tumor type, and similarly to other tumors, from several studies it has emerged that the expression of PSMA is exclusive to new vessels and not to tumor cells, suggesting possible roles in neurotransmission and carcinogenesis [37]. Wernicke et al., through an IHC analysis, showed that 100% of 32 paraffin-embedded samples consisting of glioblastoma vessels showed PSMA expression. In most cases (91%), the intensity was moderate or intense [52]. Conversely, a study conducted by Saffar et al., also corroborated by an IHC analysis, showed that only 15 out of 36 gliomas (12 of high grade and 3 of low grade) showed a positive reaction to PSMA [53]. This discrepancy may be due to the use of different clones of antibodies targeting different antigenic epitopes and may depend on the histology and grade. Indeed, as it also emerged from the review published by Bertagna et al., glioblastomas are more PSMA-avid than gliomas, offering the opportunity to be studied with PSMA-based PET/CT imaging as an alternative to ^18^F-FLT, and overcoming the limitations of ^18^F-FDG in studying brain tumors [37,54,55].

PSMA expression in glioblastomas could also open new possibilities for treatment, but at the moment ^177^Lu-PSMA has been attempted in only two patients [56,57] (Table 1). The low uptake in normal cerebral parenchyma and consequently the very high T/B ratio observed via PSMA PET imaging are great reasons to use ^177^Lu-based agents to avoid high-grade radiotoxicity to the surrounding normal parenchyma, which can occur after external beam radiotherapy. Kunikowska et al. evaluated ^68^Ga-PSMA-11 uptake in 15 patients affected by glioblastoma multiforme, reporting that 40% of them had a tumor/liver ratio > 1 and 13% had a tumor/liver ratio > 1.5. This would suggest, in accordance with the current indication for PRLT with ^177^Lu-PSMA [26], a possible beneficial role of PSMA in some of these patients [55]. The same authors also described the first case report of a patient with glioblastoma recurrence treated with a single dose of ^177^Lu-PSMA (8.4 GBq). The serial SPECT imaging showed the increasing uptake of ^177^Lu-PSMA over time, but the authors did not mention the clinical outcome for the patient [56]. Kumar et al. described a patient who was pre-treated with surgery, radiotherapy and temozolomide and who underwent 3 cycles of ^177^Lu-PSMA (each 3.7 GBq); the restaging MRI revealed tumor shrinkage and minimal residual enhancement. Moreover, the patient experienced symptom relief and an improved quality of life, indicating a partial response to therapy [57].

## 7. Breast Cancer

Breast cancer is the most frequent malignancy among women (and the second most common cancer overall), but it is only at 5th place as a cause of death, thanks to its quite favourable prognosis, mainly due to advances in of screening, diagnosis and therapy. However, the prognosis may be poor in some histotypes, such as in triple-negative breast cancer, due to the lack of targeted therapies. Therefore, great efforts have been devoted to discovering new therapeutic strategies. Similarly to other tumors, the presence of PSMA has been mainly observed in the neovasculature. Tolkach et al. studied 315 patients affected by breast cancer with IHC, reporting PSMA expression in the tumoral endothelia in 189 samples (60%) [58]. Interestingly, they also found additional weak PSMA expression in epithelial cancer cells in 10 samples. The highest PSMA levels were observed in higher grades, in hormone-receptor-negative, triple-negative cancers and Her2-positive cancers. They also reported a case of a 38-year-old patient affected by triple-negative breast cancer, who did not respond to standard therapies and was treated with PSMA-RLT, based on a positive ^68^Ga-PSMA PET/CT. The patient received 2 cycles of 7.5 GBq of ^177^Lu-PSMA-RLT every 4 weeks without any side-effect and with the evidence, at post-therapy SPECT imaging, of high uptake in tumor lesions. However, important disease progression was observed after the second cycle and the treatment was stopped [58]. Wernicke et al. described the endothelial expression of PSMA in 90 out of 92 investigated breast cancer patients, reporting PSMA positivity also in distant metastases, with the same intensity of the primary tumor [59]. Interestingly, in another large series studied by Kasoha et al., PSMA expression was detected in 72% of cases in tumor cells, and only in 46% in the tumor-associated neovasculature. Moreover, 39 normal breast tissue samples were also examined and showed the presence of the antigen in the glandular breast cells but not in the normal vessels [60].

Concerning ^68^Ga-PSMA PET/CT imaging in breast cancer, Sathekge et al. published one of the largest series, enrolling 19 patients with new diagnosed breast carcinoma. Eighty-one out of 94 lesions were identified, with an overall detection rate of 84%, since 6 primary or recurrent lesions, 2 lymph-nodes and 5 other distant metastases were not detected. Interestingly, the SUV values showed a wide variation, not only amongst different patients, but also between different lesions of the same patient. They also performed ^18^FDG PET/CT scans in 7 patients and identified 35 positive lesions, of which six were PSMA-negative. Only one lesion was PSMA-positive and ^18^FDG-negative. Taken together, their results support the concept of the spatial and temporal heterogeneity of the PSMA expression in primary tumors and distant metastases [61].

Another study, published by Medina-Ornelas et al., compared ^18^FDG and ^68^Ga-PSMA PET/CT in 21 patients with breast cancer, reporting lower detection rates for ^68^Ga in patients with hormones-positive receptors and a comparable detection rate in triple-negative patients, supporting the possible role of ^68^Ga-PSMA PET/CT in the risk stratification and identification of the aggressiveness of the disease [62]. Nevertheless, for breast carcinoma, as well as for other non-prostatic tumors, more evidences are needed to definitively collocate this approach in the diagnostic workup, and most important, other studies should investigate the possible application of ^177^Lu-PSMA RLT. Indeed, to the best of our knowledge, RLT has been attempted in only one patient [58] (Table 1). Nevertheless, in a preclinical study, ^177^Lu-PSMA RLT proved to significantly impair the vitality and the angiogenic properties of endothelial cells of breast cancer, representing a potential future therapeutic option in non-responder patients [63].

## 8. Lung Cancer

**Figure 3:**
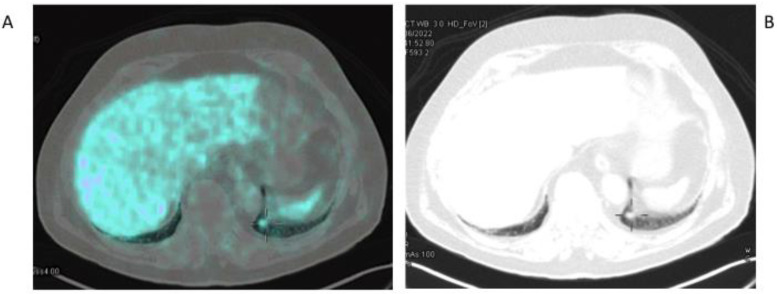
Incidental finding of primary lung cancer in a 72-year-old patient studied with ^68^Ga-PSMA-11 for prostate cancer; the PSA levels at the time of the study were 0.005 ng/mL. A fused PET/CT image (**A**) showing mild ^68^Ga-PSMA uptake in the posterior basal segment of the left lung corresponding to a small lung nodule from a co-registered low-dose CT scan (**B**). Histology showed a primary lung adenocarcinoma.

Lung cancer is the first cause of cancer incidence and mortality worldwide, being the second most common cancer diagnosed in men (after PCa) and women (after breast cancer) [64,65]. It is usually classified in two histological groups, small-cell lung cancer (SCLC) and non-small cell lung cancer (NSCLC), which includes adenocarcinoma, squamous cell carcinoma and large cell carcinoma, each one with a specific sub-classification [66]. Since PCa and lung cancer are the most frequent tumors in men, several case reports in the literature have described the incidental detection of positive lung findings on ^68^Ga-PSMA PET/CT conducted in patients with PCa, which were confirmed to be primary lung cancers based on the histology results [67,68,69,70] (Figure 3).

Pyka et al., in particular, retrospectively evaluated 89 PSMA-positive lung lesions detected in 45 patients with PCa with ^68^Ga-PSMA PET/CT. Histology confirmed PCa metastases in 39 lesions, 37 were highly probable PCa metastases, in 2 cases an activated tuberculosis was detected, 7 lesions were classified as lung cancer and 4 lesions remained unclear. Although the SUVmax in lung cancer was higher than in PCa metastases (5.6 vs. 4.4), this difference was not significant, showing that the use of a semi-quantitative analysis, using SUVmax, is not reliable in differentiating lung PCa metastases from primary tumors [71]. The same conclusion was reached by Osman et al., who retrospectively evaluated 764 patients with PCa and 49 possible synchronous malignant tumors (not only pulmonary), underlining that isolated PSMA-positive lung lesions in PCa patients should always be interpreted with caution, via a correlation with clinical and biochemical information (e.g., PSA levels), and may often require a histology confirmation or further imaging examinations, such as ^18^F-FDG PET/CT [70].

However, to the best of our knowledge, no comparative studies on ^18^F-FDG and PSMA uptake in patients with lung cancer have been published in the literature. In a case report, the authors described a lung-speculated lesion showing intense ^68^Ga-PSMA uptake and no pathological ^18^F-FDG uptake [72]. Histology performed after lobectomy revealed a highly differentiated primary adenocarcinoma of the lung. Despite the amply recognized important role of ^18^F-FDG PET/CT in the evaluation of solitary pulmonary nodules and in the staging of lung cancer patients, ^18^F-FDG is not free from pitfalls, and this report showed that some selected cases (e.g., histological non-FDG avid subtypes) could benefit from an alternative “dual-tracer” approach.

Wang et al. performed an IHC analysis in 87 NSCLC patients and 30 SCLC patients, reporting PSMA expression in the neovasculature in 85% of NSCLC cases, which was clearly correlated to the clinical stage: patients at stages I and II presented higher percentages of PSMA expression compared to patients at stage III. This was probably due to the greater presence of the neovasculature in early-stage patients rather than late-stage tumors, which are mainly characterized by necrosis. Moreover, they also detected PSMA expression in the neovessels of 70% of patients affected by SCLC, without any correlation with the clinical stage. Interestingly, they found that PSMA was also expressed by tumor cells of NSCLC patients (54% of cases) [73]. These findings were not confirmed by Schmidt et al., who investigated via IHC 275 resected NSCLC patients, reporting a great amount of PSMA expression in the endothelium of the tumor vessels (49% of cases) but a lower fraction in tumor cells (6%), mainly in the squamous cancer subtype (82%). They did not find any prognostic correlation between the PSMA expression and OS, but the neovasculature PSMA expression was associated with a higher histologic grade (G3/G4), probably due to the relatively higher intra-tumoral hypoxia [74].

At the moment, no case reports on lung cancer treated with PSMA-RLT have been published yet, but the encouraging results reached so far will open new perspectives and future clinical applications in this field.

## 9. Other Cancers

Following the incidental findings reported using ^68^Ga-PSMA PET/CT, PSMA expression has been also evaluated in many other solid tumors, and in particular in gastric cancers (GC) and colorectal carcinomas (CRC) [75,76,77,78,79,80,81]. On the basis of these reports, Haffner et al. conducted an IHC analysis in 199 patients who underwent surgery for gastric adenocarcinoma, reporting PSMA expression on tumor neovessels in 66.4% of cases and in 130 patients surgically treated for CRC, including 19 liver and 5 lymph node metastases, reporting endothelial PSMA expression in 84.6%, 64.2% and 80% of cases, respectively. In patients with both primary tumors and metastases, they found the same expression pattern and score, concluding that PSMA expression is conserved even in disease progression [82]. Abdel-Hadi et al. reached similar results, reporting positive PSMA staining in the neovasculature of 75 out of 100 CRCs analyzed and finding a statistically significant correlation between the PSMA expression, distant metastases and vascular invasion [83]. No clinical experiences have been reported for ^177^Lu-RLT therapy in cases of CRC, but Cuda et al. [84] analyzed the possible role of semi-quantitative analyses in order to asses whether metastatic patients affected by CRC could meet the criteria to predict their response to RLT, as recently used for the TheraP trial criteria (SUVmax of at least 10 in all tumor sites, SUVmax of more than 20 at the most avid site and ^68^Ga-PSMA-11 avidity higher than ^18^F-FDG avidity in all sites) [85]; unfortunately, none of the 10 patients evaluated with ^18^F-FDG and ^68^Ga-PSMA PET/CT met these criteria, showing consistently lower or absent PSMA uptake in the lesions compared to ^18^F-FDG [84].

Sarcomas have also been investigated using ^68^Ga-PSMA PET/CT and IHC, and several authors have reported a correlation between PSMA expression and the disease’s aggressiveness. Zeng et al., using IHC, analyzed 45 cases of osteosarcoma and 12 of osteofibrous dysplasia (OD), reporting that while no cases of OD were positive for PSMA immune staining, 46% of osteosarcoma cases showed PSMA expression on the endothelial cells associated with the tumor, and this was significantly associated with the tumor size, the risk of developing pulmonary metastases and the worst clinical outcome, suggesting that PSMA expression could be considered a prognostic marker [86]. Sasikumar et al. reported a case of osteosarcoma developing in fibrodysplasia evaluated with ^68^Ga-PSMA PET/CT, which showed selective uptake in malignant transformation areas [87]. Heitkötter et al. evaluated PSMA staining in 779 soft tissue or bone-benign and malignant tumors (ganglioneuromas, myxofibrosarcomas, extrapleural solitary fibrous tumors, inflammatory myofibroblastic tumors, desmoid-type fibromatosis, Ewing sarcomas, gastrointestinal stromal tumors, synovial sarcomas, undifferentiated pleomorphic sarcomas and endometrial stromal sarcomas), reporting that endothelial PSMA expression was more frequent in malignant tumors than in tumors with intermediate or benign biological behaviour; in addition, they described a case of alveolar rhabdomyosarcoma with very strong PSMA expression in tumor cells [88]. Differing from the majority of mesenchymal tumors, which express PSMA exclusively in tumor neovessels, Vaz et al. described a case of gastrointestinal stromal tumors (GIST) with intense uptake of PSMA based on PET imaging and a high expression level of PSMA in the membrane and cytoplasm of cancer cells [89].

Similar results have been reported for pancreatic cancers. Several incidental positive findings in the pancreatic gland have been described using ^68^Ga-PSMA PET/CT in patients affected by PCa, which were found to be primary pancreatic tumors [90,91,92]. In these and other studies, even if in a smaller percentage, PSMA expression was detected also on the tumor cells and not only in neovessels [93,94,95]. Ren et al. found positive correlations between PSMA expression, OS and several clinical–pathological parameters of pancreatic adenocarcinoma (such as the histological grade and pTNM stage). Moreover, performing PSMA PET/CT could be of value in pancreatic cancers when conventional imaging, with CT or MRI, may show equivocal findings and when some inflammatory lesions can mimic malignancy when using ^18^F-FDG PET/CT [94]. In particular, Krishnaraju et al. compared ^68^Ga-PSMA and ^18^F-FDG PET/CT results in a patient with a newly diagnosed pancreatic mass, reporting high overall sensitivity for both radiopharmaceuticals (94.7% vs. 89.5%) and higher specificity for PSMA (90% vs. 57.1%) for the detection of the primary pancreatic tumor; the authors concluded that ^68^Ga-PSMA PET/CT imaging may be more accurate in the pre-operative setting, when an early diagnosis and surgical resection can strongly improve the clinical outcome of the patients with localized disease [96].

Regarding the urinary system, PSMA expression was detected in the normal bladder urothelium, although it was much weaker than in prostate-derived tissues, and in different subtypes of bladder carcinoma (such as urothelial carcinoma, squamous cell carcinoma, adenocarcinoma and small cell carcinoma), either on the endothelial cells of tumor vessels or on tumor cells [97,98]. Despite different studies reporting the variable expression of PSMA, it seems that the rate of expression is relatively low compared to PCa and the other tumors mentioned above. Although some studies reported the metastasis of urothelial carcinoma being PSMA-avid in PET images [99], Campbell et al. described a case series of three patients with metastatic urothelial carcinoma evaluated using ^18^F-DCFPyL PET/CT and IHC, showing low uptake in metastatic sites and low levels or nearly absent PSMA expression in tumor neovessels [100].

PSMA expression has been also investigated in gynecological malignancies, in particular in ovarian, endometrial, cervical and vulvar carcinomas, but the data reported in the ovarian cancer setting are conflicting. Wernicke et al. detected PSMA expression in the tumor vessels of all the 21 ovarian cancer samples analyzed and in 10 cases on the tumor cells; they also compared the PSMA expression levels in metastatic tumor foci, showing higher or similar rates of PSMA-positive capillaries than their respective primary ovarian tumors [101]; similar results have been reported by Hofstetter et al., who found PSMA expression in the great majority of ovarian cancer samples and suggesting a direct correlation with OS [102]. Conversely, Aide et al., studying 32 patients affected by serous ovarian cancers, reported very weak PSMA expression levels in the entire dataset, concluding that PSMA cannot be used as a prognostic marker or a target for a theranostic strategy in this setting [103].

Finally, PSMA expression on the endothelium of tumoral vessels has been detected in many other different tumors, including more common histotypes, such as oral squamous cell carcinomas [104,105] and melanomas [106], and relatively rare neoplasms, such as endolymphatic sac tumors [107].

To the best of our knowledge, regarding the application of ^177^Lu-PSMA RLT, no other cases, in addition to those mentioned above, have been reported, with the exception of one patient, described by Simsek et al., who was affected by a progressive metastatic testicular mixed germ cell tumor, who after ^177^Lu-PSMA RLT achieved a mixed therapy response based on the ^68^Ga-PSMA PET/CT imaging results but showed a concomitant increased level of alfa-feto protein [108] (Table 1). However, different approaches able to target PSMA in solid tumors have been evaluated and are still under investigation, such as using monoclonal antibodies [109] or compounds conjugated with PSMA ligands [110], but more evidence is needed to assess whether they can be reliable tools in clinical practice.

## 10. Is There an Added Value of PSMA-Based Imaging over ^18^F-FDG PET/CT for Extra-Prostatic Tumors?

^18^F-FDG is one of the most commonly used radiopharmaceuticals for PET imaging, and for many years it played a crucial role in the staging and restaging of several cancers. Nevertheless, with the advances in knowledge of tumor biology and heterogeneity and with the quick progress in the radiochemistry field, the unmet clinical need emerged to better characterize some tumors that are “orphans” of a NM modality of choice or where ^18^F-FDG has only a limited role, and to better select patients eligible for targeted therapy. Therefore, novel and more specific SPECT and PET radiopharmaceuticals have been developed as alternatives to ^18^F-FDG. In this setting, PSMA-based imaging is demonstrating to be a promising approach for imaging several cancers. This radiopharmaceutical is providing an alternative tool, not only for imaging cancer tissues that are hidden by the physiologic biodistribution of ^18^F-FDG (such as brain or liver tumors), but also to allow a prompt evaluation of these cancers before their de-differentiation and the consequent appearance of ^18^F-FDG-avid lesions, being potentially useful for therapy decision making and for planning personalized treatments.

The diagnostic performances of PSMA-based imaging and ^18^F-FDG have been compared by several authors, mainly in case reports, in thyroid cancer [20,21,22,23,111,112,113,114,115,116], adenoid cystic carcinoma [14], HCC [34], lung cancer [72], gastric carcinoma [75], colorectal cancer [84], pancreatic tumors [96], renal cell carcinomas [44,45,46,48], urothelial carcinoma [100,117], Ewing sarcoma [118] and metastatic osterosarcoma [119].

Taken together, a complementary role of these two modalities has emerged to look at the same phenomenon from different points of view, providing molecular information on tumor biology. The evidence of PSMA expression by a tumor would offer the possibility to treat patient with RLT; therefore, some patients would benefit from a dual-tracer approach. Nevertheless, further prospective comparative studies on larger series are needed to assess whether PSMA PET imaging could be included in the diagnostic work-up of oncological diseases, and in which histotypes and in which phases of the disease it could be appropriate to perform a PSMA study.

## 11. Conclusions and Future Perspectives

In the past decades, the body of literature regarding non-prostatic benign and malignant diseases exhibiting PSMA uptake based on PET imaging has become more and more consistent. Although these findings have raised the problem of the relatively low specificity of PSMA, on the other hand, this aspect has allowed the discovery of new insights on tumor biology and is opening up new interesting and alternative diagnostic and therapeutic opportunities. From the preliminary reports, indeed PSMA is emerging as an additional theranostic tool that could be used to select patients eligible for ^177^Lu-PSMA therapies, especially in those cancers where conventional imaging and treatments may fail. Nevertheless, larger prospective studies exploring PSMA PET/CT and ^177^Lu-PSMA RLT in non-prostate cancers are the key in the assessment of their possible application in clinical practice, but at the moment these studies are still lacking. Several clinical trials (https://clinicaltrials.gov/ct2/search accessed on 21 October 2022) aiming to assess the role of PSMA-based imaging in HCC (NCT05547919; NCT05580835), gastric cancer (NCT05214820), salivary gland tumors (NCT05581979) and renal cell carcinomas (NCT04987086) are ongoing. Nevertheless, to the best of our knowledge, only a few are available on the use of ^177^Lu-PSMA RLT in extra-prostatic tumors (NCT04291300; NCT05170555).

Preclinical studies are also needed to evaluate the therapeutic effects of PSMA-RLT in non-prostatic malignancies in order to evaluate ^177^Lu-PSMA retention in tumors where PSMA is expressed only in the neovasculature, since a different PSMA localization might influence the kinetics of the therapeutic agent. Finally, the eligibility criteria for ^177^Lu-PSMA RLT in other tumors should be defined, as has already be done for prostate glands.

The way towards the implementation of PSMA-based imaging and therapy in clinical practice seems still far away, but research is going fast in this direction and is providing very promising and encouraging perspectives. We can, therefore, expect that this molecule will open new theranostic possibilities in the near future.

**Table 1:** Patients with non-prostate cancer treated with PSMA RLT.

Cancer Type	Histotype (n. of pts)	PSMA PET Findings (n. of pts)	Criteria for Eligibility to RLT	Dose × n. Cycles (n. of pts)	Clinical/Biochemical Response (n. of pts)	Radiological Response (n. of pts)	Reference
*Salivary gland cancer*	AdCC (4)	Lung metastases (2) Liver metastases (1) Bone metastases (1) Locoregional recurrence (1)	PSMA uptake > normal liver on PET imaging	6–7.4 GBq × 2 (2) 6–7.4GBq × 4 (2)	Symptom relief (3) PD (1)	Stable lung lesions, minimal progression of liver metastases (1) SD (1) PD (2)	[15]
	Adenocarcinoma NOS (1)	Bone metastases	PSMA uptake > normal liver on PET imaging	6–7.4 GBq × 1	Treatment was discontinued due to side-effect development (fatigue, xerostomia)	N.A.	[15]
	Acinar cell carcinoma (1)	Inguinal lymph nodes Lung metastases Bone metastases	PSMA uptake > normal liver on PET imaging	6–7.4 GBq × 2	Significant pain relief but treatment was discontinued due to side-effect development (fatigue, xerostomia)	PD	[15]
*Thyroid cancer*	RrDTC (1)	Neck and lung metastases	PSMA+ lesions (no other criteria for eligibility were specified)	7.4 GBq × 1	N.A. Patient died after 2 weeks due to cardiac arrest	N.A.	[27]
	RrDTC (2)	Neck metastases (2) Lung metastases (2) Liver metastases (1)	PSMA+ lesions (no other criteria for eligibility were specified)	6 GBq × 2	Slightly temporary (7 months) decrease in Tg levels (1) Increase in Tg levels (1)	PR (1) PD (1)	[21]
	PDTC (1)	Bone metastases	PSMA+ lesions (no other criteria for eligibility were specified)	6.3 Gbq × 1 and 7.4 Gbq × 1	Temporary (7 months) stable Tg levels	Temporary SD followed by appearance of FDG positive lesions	[20]
*Liver cancer*	HCC (2)	Liver lesions (2)	PSMA+ lesions (no other criteria for eligibility were specified)	5.9–6.2 GBq × 1	N.A. Treatment was discontinued due to poor dosimetry at SPECT/CT	N.A.	[35]
*Brain cancer*	GBM (1)	Right parietal mass	PSMA uptake > normal liver on PET imaging in patients not eligible for surgery or who refused RT/CHT	8.4 GBq × 1	N.A.	Increased uptake on SPECT imaging after therapy but no radiological FU was reported	[56]
	GBM (1)	Left temporal nodular lesion extending to left gangliocapsular region	PSMA uptake > normal liver on PET imaging in patients not eligible for surgery or who refused RT/CHT	3.7 GBq × 3	Improvements in QoL and pain relief	Significant reduction in lesion size at restaging MRI	[57]
*Breast cancer*	Triple negative breast cancer (1)	Local recurrence and metastatic lymph nodes	PSMA+ lesions (no other criteria for eligibility were specified)	7.5 GBq × 2	PD	PD after the second cycle	[58]
*Testicular Cancer*	Testicular mixed germ cell tumor (1)	Bone, liver, lymph nodes, lung metastases	PSMA+ lesions (no other criteria for eligibility were specified)	7.5 GBq × 1	Increased α-fetoprotein levels	PD	[108]

List of abbreviations: RLT: radioligand therapy; GBq: gigabequerel; N.A.: not available; SD: stable disease; PD: progression disease; PR: partial response; AdCC: adenocistic carcinoma; NOS: not otherwise specified; RrDTC: radioiodine-refractory differentiated thyroid cancer; PTC: papillary thyroid carcinoma; PDTC: poorly differentiated thyroid carcinoma; Tg: tireoglobulin; HCC: hepatocellular carcinoma; GBM: glioblastoma multiforme; FU: follow-up; RT: radiotherapy; CHT: chemotherapy; QoL: quality of life; MRI: magnetic resonance imaging.

## Data Availability

Not applicable.

## References

[1] Denise S., O’Keefe D.S., Bacich D.J., Huang S.S., Heston W.D.W. (2018). A perspective on the evolving story of PSMA biology, PSMA-based imaging and endoradiotherapeutic strategies. J. Nucl. Med..

[2] Eiber M., Fendler W.P., Rowe S.P., Calais J., Hofman M.S., Maurer T., Schwarzenboeck S.M., Kratowchil C., Herrmann K., Giesel F.L. (2017). Prostate-specific membrane antigen ligands for imaging and therapy. J. Nucl. Med..

[3] Fragomeni R.A.S., Amir T., Sheikhbahaei S., Harvey S.C., Javadi M.S., Solnes L.B., Kiess A.P., Allaf M.E., Pomper M.G., Gorin M.A. (2018). Imaging of nonprostate cancers using PSMA-targeted radiotracers: Rationale, current state of the field, and a call to arms. J. Nucl. Med..

[4] Siva S., Udovicich C., Tran B., Zargar H., Murphy D.G., Hofman M.S. (2020). Expanding the role of small-molecule PSMA ligands beyond PET staging of prostate cancer. Nat. Rev. Urol..

[5] Lutje S., Heskamp S., Cornelissen A.S., Poeppel T.D., van den Broek S.A., Rosenbaum-Krumme S., Bockisch A., Gotthardt M., Rijpkema M., Boerman O.C. (2015). PSMA ligands for radionuclide imaging and therapy of prostate cancer: Clinical status. Theranostics.

[6] Richter S., Wuest F. (2014). 18F-Labeled peptides: The future is bright. Molecules.

[7] Ducharme J., Goertzen A.L., Patterson J., Demeter S. (2009). Practical aspects of 18F-FDG PET when receiving 18F-FDG from a distant supplier. J. Nucl. Med. Technol..

[8] De Galiza Barbosa F., Queiroz M.A., Nunes R.F., Costa L.B., Zaniboni E.C., Marin J.F.G., Cerri G.G., Buchpiguel C.A. (2020). Nonprostatic diseases on PSMA PET imaging: A spectrum of benign and malignant findings. Cancer Imaging.

[9] Rupp N.J., Umbricht C.A., Pizzuto D.A., Lenggenhager D., Töpfer A., Müller J., Muehlematter U.J., Ferraro D.A., Messerli M., Morand G.B. (2019). First clinicopathologic evidence of a Non-PSMA-Related uptake mechanism for (68)Ga-PSMA-11 in salivary glands. J. Nucl. Med..

[10] Nishida H., Kondo Y., Kusaba T., Kadowaki H., Daa T. (2022). Immunohistochemical reactivity of prostate-specific membrane antigen in salivary gland Tumors. Head Neck Pathol..

[11] Van Boxtel W., Lütje S., van Engen-van Grunsven I.C.H., Verhaegh G.W., Schalken J.A., Jonker M.A., Nagarajah J., Gotthardt M., van Herpen C.M.L. (2020). ^68^Ga-PSMA-HBED-CC PET/CT imaging for adenoid cystic carcinoma and salivary duct carcinoma: A phase 2 imaging study. Theranostics.

[12] Klein Nulent T.J.W., van Es R.J.J., Krijger G.C., de Bree R., Willems S.M., de Keizer B. (2017). Prostate-specific membrane antigen PET imaging and immunohistochemistry in adenoid cystic carcinoma-a preliminary analysis. Eur. J. Nucl. Med. Mol. Imaging.

[13] Konig L., Hauswald H., Flechtenmacher C., Heller M., Debus J., Haberkorn U., Kratochwil l., Giesel F. (2017). Uptake of prostate-specifc membrane antigen (PSMA) in adenoid cystic carcinoma—Is PSMA-PET-CT a helpful tool in radiation oncology?. Clin. Transl. Radiat. Oncol..

[14] Isgoren S., Hekimsoy T., Koroglu E., Demir H. (2022). PET/CT with 68Ga-PSMA and 18F-FDG in Metastatic Adenoid Cystic Carcinoma: Report of 2 Cases. Clin. Nucl. Med..

[15] Klein Nulent T.J.W., van Es R.J.J., Willems S.M., Braat A.J.A.T., Devriese L.A., de Bree R., de Keizer B. (2021). First experiences with 177Lu-PSMA-617 therapy for recurrent or metastatic salivary gland cancer. EJNMMI Res..

[16] Roman B.R., Morris L.G., Davies L. (2017). The thyroid cancer epidemic, 2017 perspective. Curr. Opin. Endocrinol. Diabetes Obes..

[17] Luster M., Clarke S.E., Dietlein M., Lassmann M., Lind P., Oyen W.J., Tennvall J., Bombardieri E. (2008). Guidelines for radioiodine therapy of differentiated thyroid cancer. EJNMMI.

[18] Bertagna F., Albano D., Giovanella L., Bonacina M., Durmo R., Giubbini R., Treglia G. (2019). 68Ga-PSMA PET thyroid incidentalomas. Hormones.

[19] Bychkov A., Vutrapongwatana U., Tepmongkol S., Keelawat S. (2017). PSMA expression by microvasculature of thyroid tumors–Potential implications for PSMA theranostics. Sci. Rep..

[20] Wächter S., Di Fazio P., Maurer E., Manoharan J., Keber C., Pfestroff A., Librizzi D., Bartsch D.K., Luster M., Eilsberger F. (2021). Prostate-specific membrane antigen in anaplastic and poorly differentiated thyroid cancer—A new diagnostic and therapeutic target?. Cancers.

[21] De Vries L.H., Lodewijk L., Braat A.J., Krijger G.C., Valk G.D., Lam M.G., Rinkes I.H., Vriens M.R., de Keizer B. (2020). 68 Ga-PSMA PET/CT in radioactive iodine-refractory differentiated thyroid cancer and first treatment results with 177 Lu-PSMA-617. EJNMMI Res..

[22] Lawhn-Heath C., Yom S.S., Liu C., Villanueva-Meyer J.E., Aslam M., Smith R., Narwal M., Juarez R., Behr S.C., Pampaloni M.H. (2020). Gallium-68 prostate-specific membrane antigen ([68 Ga] Ga-PSMA-11) PET for imaging of thyroid cancer: A feasibility study. EJNMMI Res..

[23] Verma P., Malhotra G., Agrawal R., Sonavane S., Meshram V., Asopa R.V. (2018). Evidence of prostate-specific membrane antigen expression in metastatic differentiated thyroid cancer using 68Ga-PSMA-HBED-CC PET/CT. Clin. Nucl. Med..

[24] Pitalua-Cortes Q., García-Perez F.O., Vargas-Ahumada J., Gonzalez-Rueda S., Gomez-Argumosa E., Ignacio-Alvarez E., Soldevilla-Gallardo I., Torres-Agredo L. (2021). Head-to-head comparison of 68Ga-PSMA-11 and 131I in the follow-up of well-differentiated metastatic thyroid cancer: A new potential theragnostic agent. Front. Endocrinol..

[25] Aashiq M., Silverman D.A., Na’ara S., Takahashi H., Amit M. (2019). Radioiodine-refractory thyroid cancer: Molecular basis of redifferentiation therapies, management, and novel therapies. Cancers.

[26] Kratochwil C., Fendler W.P., Eiber M., Baum R., Bozkurt M.F., Czernin J., Delgado Bolton R.C., Ezziddin S., Forrer F., Hicks R.J. (2019). EANM procedure guidelines for radionuclide therapy with 177Lu-labelled PSMA-ligands (177Lu-PSMA-RLT). EJNMMI.

[27] Assadi M., Ahmadzadehfar H. (2019). 177Lu-DOTATATE and 177Lu-prostate-specific membrane antigen therapy in a patient with advanced metastatic radioiodine-refractory differentiated thyroid cancer after failure of tyrosine kinase inhibitors treatment. World J. Nucl. Med..

[28] Bray F., Ferlay J., Soerjomataram I., Siegel R.L., Torre L.A., Jemal A. (2018). Global cancer statistics 2018: GLOBOCAN estimates of incidence and mortality worldwide for 36 cancers in 185 countries. CA Cancer J. Clin.

[29] Izuishi K., Yamamoto Y., Mori H., Kameyama R., Fujihara S., Masaki T., Suzuki Y. (2014). Molecular mechanisms of [18F]fluorodeoxyglucose accumulation in liver cancer. Oncol. Rep..

[30] Sacks A., Peller P.J., Surasi D.S., Chatburn L., Mercier G., Subramaniam R.M. (2011). Value of PET/CT in the management of primary hepatobiliary tumors, part 2. Am. J. Roentgenol..

[31] Sheikhbahaei S., Werner R.A., Solnes L.B., Pienta K.J., Pomper M.G., Gorin M.A., Rowe S.P. (2019). Prostate-Specific Membrane Antigen (PSMA)-Targeted PET imaging of prostate cancer: An update on important pitfalls. Semin. Nucl. Med..

[32] Kesler M., Levine C., Hershkovitz D., Mishani E., Menachem Y., Lerman H., Zohar Y., Shibolet O., Even-Sapir E. (2019). 68Ga-PSMA is a novel PET-CT tracer for imaging of hepatocellular carcinoma: A prospective pilot study. J. Nucl. Med..

[33] Kuyumcu S., Has-Simsek D., Iliaz R., Sanli Y., Buyukkaya F., Akyuz F., Turkmen C. (2019). Evidence of prostate-specific membrane antigen expression in hepatocellular carcinoma using 68Ga-PSMA PET/CT. Clin. Nucl. Med..

[34] Gündoğan C., Ergül N., Çakır M.S., Kılıçkesmez Ö., Gürsu R.U., Aksoy T., Çermik T.F. (2021). 68Ga-PSMA PET/CT versus 18F-FDG PET/CT for imaging of hepatocellular carcinoma. Mol. Imaging Radionucl. Ther..

[35] Hirmas N., Leyh C., Sraieb M., Barbato F., Schaarschmidt B.M., Umutlu L., Nader M., Wedemeyer H., Ferdinandus J., Rischpler C. (2021). 68Ga-PSMA-11 PET/CT improves tumor detection and impacts management in patients with hepatocellular carcinoma. J. Nucl. Med..

[36] Capitanio U., Bensalah K., Bex A., Boorjian S.A., Bray F., Coleman J., Gore J.L., Sun M., Wood C., Russo P. (2019). Epidemiology of renal cell carcinoma. Eur. Urol..

[37] Uijen M.J.M., Derks Y.H.W., Merkx R.I.J., Schilham M.G.M., Roosen J., Privé B.M., van Lith S.A.M., van Herpen C.M.L., Gotthardt M., Heskamp S. (2021). PSMA radioligand therapy for solid tumors other than prostate cancer: Background, opportunities, challenges, and first clinical reports. Eur. J. Nucl. Med. Mol. Imaging.

[38] Gorin M.A., Rowe S.P., Hooperc J.E., Kates M., Hammers HJSzabo Z., Pomper M.G., Allaf M.E. (2017). PSMA-targeted 18F-DCFPyL PET/CT imaging of clear renal cell carcinoma: Results from a rapid autopsy. Eur. Urol..

[39] Spatz S., Tolkach Y., Jung K., Stephan C., Busch J., Ralla B., Rabien A., Feldmann G., Brossart P., Bundschuh R.A. (2018). Comprehensive evaluation of prostate specific membrane antigen expression in the vasculature of renal tumors: Implications for imaging studies and prognostic role. J. Urol..

[40] Sawicki L.M., Buchbender C., Boos J., Giessing M., Ermert J., Antke C., Antoch G., Hautzel H. (2017). Diagnostic potential of PET/CT using a 68Ga-labelled prostate-specific membrane antigen ligand in whole-body staging of renal cell carcinoma: Initial experience. Eur. J. Nucl. Med. Mol. Imaging.

[41] Rhee H., Blazak J., Tham C.M., Ng K.L., Shepherd B., Lawson M., Preston J., Vela I., Thomas P., Wood S. (2016). Pilot study: Use of gallium-68 PSMA PET for detection of metastatic lesions in patients with renal tumour. EJNMMI Res..

[42] Evangelista L., Basso U., Maruzzo M., Novara G. (2020). The role of radiolabeled prostate-specific membrane antigen positron emission tomography/computed tomography for the evaluation of renal cancer. Eur. Urol. Focus.

[43] Rowe S.P., Gorin M.A., Hammers H.J., Som Javadi M., Hawasli H., Szabo Z., Cho S.Y., Pomper M.G., Allaf M.E. (2015). Imaging of metastatic clear cell renal cell carcinoma with PSMA-targeted ¹⁸F-DCFPyL PET/CT. Ann. Nucl. Med..

[44] Sasikumar A., Joy A., Nanabala R., Unni M., Tk P. (2016). Complimentary pattern of uptake in 18F-FDG PET/CT and 68Ga-prostate-specific membrane antigen PET/CT in a case of metastatic clear cell renal carcinoma. Clin. Nucl. Med..

[45] Saadat S., Tie B., Wood S., Vela I., Rhee H. (2018). Imaging tumour thrombus of clear cell renal cell carcinoma: FDG PET or PSMA PET? Direct in vivo comparison of two technologies. Urol. Case Rep..

[46] Rowe S.P., Gorin M.A., Hammers H.J., Pomper M.G., Allaf M.E., Javadi M.S. (2016). Detection of 18F-FDG PET/CT occult lesions with 18F-DCFPyL PET/CT in a patient with metastatic renal cell carcinoma. Clin. Nucl. Med..

[47] Siva S., Callahan J., Pryor D., Martin J., Lawrentschuk N., Hofman M.S. (2017). Utility of 68 Ga prostate specific membrane antigen-positron emission tomography in diagnosis and response assessment of recurrent renal cell carcinoma. J. Med. Imaging Radiat. Oncol..

[48] Nadebaum D.P., Hofman M.S., Mitchell C.A., Siva S., Hicks R.J. (2017). Oligometastatic renal cell carcinoma with sarcomatoid differentiation demonstrating variable imaging phenotypes on 68Ga-PSMA and 18F-FDG PET/CT: A case report and review of the literature. Clin. Genitourin. Cancer.

[49] Demirci E., Ocak M., Kabasakal L., Decristoforo C., Talat Z., Halaç M., Kanmaz B. (2014). 68Ga-PSMA PET/CT imaging of metastatic clear cell renal cell carcinoma. Eur. J. Nucl. Med. Mol. Imaging.

[50] Yin Y., Campbell S.P., Markowski M.C., Pierorazio P.M., Pomper M.G., Allaf M.E., Rowe S.P., Gorin M.A. (2019). Inconsistent detection of sites of metastatic non-clear cell renal cell carcinoma with PSMA-Targeted [18F]DCFPyL PET/CT. Mol. Imaging Biol..

[51] Omuro A., De Angelis L.M. (2013). Glioblastoma and other malignant gliomas: A clinical review. JAMA.

[52] Wernicke A.G., Edgar M.A., Lavi E., Liu H., Salerno P., Bander N.H., Gutin P.H. (2011). Prostate-specific membrane antigen as a potential novel vascular target for treatment of glioblastoma multiforme. Arch. Pathol. Lab. Med..

[53] Saffar H., Noohi M., Tavangar S.M., Saffar H., Azimi S. (2018). Expression of Prostate-Specific Membrane Antigen (PSMA) in brain glioma and its correlation with tumor grade. Iran J. Pathol..

[54] Bertagna F., Albano D., Cerudelli E., Gazzilli M., Giubbini R., Treglia G. (2020). Potential of radiolabeled PSMA PET/CT or PET/MRI diagnostic procedures in gliomas/glioblastomas. Curr. Radiopharm..

[55] Kunikowska J., Kuliński R., Muylle K., Koziara H., Królicki L. (2020). 68Ga-Prostate-specific membrane antigen-11 PET/CT: A new imaging option for recurrent glioblastoma multiforme?. Clin. Nucl. Med..

[56] Kunikowska J., Charzyńska I., Kuliński R., Pawlak D., Maurin M., Królicki L. (2020). Tumor uptake in glioblastoma multiforme after IV injection of [177Lu] Lu-PSMA-617. Eur. J. Nucl. Med. Mol. Imaging.

[57] Kumar A., Ballal S., Yadav M.P., ArunRaj S.T., Haresh K.P., Gupta S., Damle N.A., Garg A., Tripathi M., Bal C. (2020). 177Lu-/68Ga-PSMA theranostics in recurrent glioblastoma multiforme: Proof of concept. Clin. Nucl. Med..

[58] Tolkach Y., Gevensleben H., Bundschuh R., Koyun A., Huber D., Kehrer C., Hecking T., Keyver-Paik M.D., Kaiser C., Ahmadzadehfar H. (2018). Prostate-specific membrane antigen in breast cancer: A comprehensive evaluation of expression and a case report of radionuclide therapy. Breast Cancer Res. Treat..

[59] Wernicke A.G., Varma S., Greenwood E.A., Christos P.J., Chao K.S.C., Liu H., Bander N.H., Shin S.J. (2014). Prostate-specific membrane antigen expression in tumor-associated vasculature of breast cancers. Apmis.

[60] Kasoha M., Unger C., Solomayer E.F., Bohle R.M., Zaharia C., Khreich F., Wagenpfeil S., Juhasz-Böss I. (2017). Prostate-specific membrane antigen (PSMA) expression in breast cancer and its metastases. Clin. Exp. Metastasis.

[61] Sathekge M., Lengana T., Modiselle M., Vorster M., Zeevaart J., Maes A., Ebenhan T., Van de Wiele C. (2017). 68Ga-PSMA-HBED-CC PET imaging in breast carcinoma patients. Eur. J. Nucl. Med. Mol. Imaging.

[62] Medina-Ornelas S., García-Perez F., Estrada-Lobato E., Ochoa-Carrillo F. (2020). 68Ga-PSMA PET/CT in the evaluation of locally advanced and metastatic breast cancer, a single center experience. Am. J. Nucl. Med. Mol. Imaging.

[63] Morgenroth A., Tinkir E., Vogg A.T.J., Sankaranarayanan R.A., Baazaoui F., Mottaghy F.M. (2019). Targeting of prostate-specific membrane antigen for radio-ligand therapy of triple-negative breast cancer. Breast Cancer Res..

[64] Thandra K.C., Barsouk A., Saginala K., Aluru J.S., Barsouk A. (2021). Epidemiology of lung cancer. Contemp. Oncol. Współczesna Onkol..

[65] Hirsch F.R., Scagliotti G.V., Mulshine J.L., Kwon R., Curran W.J., Wu Y.L., Paz-Ares L. (2017). Lung cancer: Current therapies and new targeted treatments. Lancet.

[66] Zheng M. (2016). Classification and pathology of lung cancer. Surg. Oncol. Clin..

[67] Chia J.Y., Loi H.Y., Khor L.K., Lee K.C., Seow Y.H. (2018). Primary lung adenocarcinoma with 68gallium prostate-specific membrane antigen-PET/CT scan avidity in a patient on surveillance after prostatectomy. Clin. Genitourin. Cancer.

[68] Sharma P. (2020). 68Ga-PSMA-Avid small cell lung cancer on PET/CT: Incidental second malignancy in treated prostate cancer. Clin. Nucl. Med..

[69] Karyağar S.S. (2020). PSMA-positive secondary tumors in 68Ga-PSMA PET/CT imaging in patients with prostate cancer. Eur. Arch. Med. Res..

[70] Osman M.M., Iravani A., Hicks R.J., Hofman M.S. (2017). Detection of synchronous primary malignancies with 68Ga-labeled prostate-specific membrane antigen PET/CT in patients with prostate cancer: Frequency in 764 patients. J. Nucl. Med..

[71] Pyka T., Weirich G., Einspieler I., Maurer T., Theisen J., Hatzichristodoulou G., Schwamborn K., Schwaiger M., Eiber M. (2016). 68Ga-PSMA-HBED-CC PET for differential diagnosis of suggestive lung lesions in patients with prostate cancer. J. Nucl. Med..

[72] Jochumsen M.R., Gormsen L.C., Nielsen G.L. (2018). 68Ga-PSMA avid primary adenocarcinoma of the lung with complementary low 18F-FDG uptake. Clin. Nucl. Med..

[73] Wang H.L., Wang S.S., Song W.H., Pan Y., Yu H.P., Si T.G., Liu Y., Cui X.N., Guo Z. (2015). Expression of prostate-specific membrane antigen in lung cancer cells and tumor neovasculature endothelial cells and its clinical significance. PLoS ONE.

[74] Schmidt L.H., Heitkötter B., Schulze A.B., Schliemann C., Steinestel K., Trautmann M., Marra A., Hillejan L., Mohr M., Evers G. (2017). Prostate specific membrane antigen (PSMA) expression in non-small cell lung cancer. PLoS ONE.

[75] Malik D., Kumar R., Mittal B.R., Singh H., Bhattacharya A., Sood A., Sharma V., Singh H. (2018). 68 Ga-labelled PSMA (prostate specific membrane antigen) expression in signet-ring cell gastric carcinoma. Eur. J. Nucl. Med. Mol. Imaging.

[76] Laurens S.T., Witjes F., Janssen M., Flucke U., Gottardt M. (2018). 68Ga-prostate-specific membrane antigen uptake in gastrointestinal stromal tumor. Clin. Nucl. Med..

[77] Sasikumar A., Joy A., Pillai M.R. (2017). 68Ga-PSMA uptake in an incidentally detected gastrointestinal stromal tumor in a case of suspected carcinoma prostate. Clin. Nucl. Med..

[78] Huang Y.T., Fong W., Thomas P. (2016). Rectal Carcinoma on 68Ga-PSMA PET/CT. Clin. Nucl. Med..

[79] Stoykow C., Huber-Schumacher S., Almanasreh N., Jilg C., Ruf J. (2017). Strong PSMA radioligand uptake by rectal carcinoma: Who put the “S” in PSMA?. Clin. Nucl. Med..

[80] Hangaard L., Jochumsen M.R., Vendelbo M.H., Bouchelouche K. (2017). Metastases from colorectal cancer avid on 68Ga-PSMA PET/CT. Clin. Nucl. Med..

[81] Sonni I., Caron J., Kishan A.U., Muthusamy V.R., Calais J. (2020). PSMA expression in the neovasculature associated with rectal adenocarcinoma: A potential stromal target for nuclear theranostics. Clin. Nucl. Med..

[82] Haffner M.C., Kronberger I.E., Ross J.S., Sheehan C.E., Zitt M., Mühlmann G., Öfner D., Zelger B., Ensinger C., Yang X.J. (2009). Prostate-specific membrane antigen expression in the neovasculature of gastric and colorectal cancers. Hum. Pathol..

[83] Abdel-Hadi M., Ismail Y., Younis L. (2014). Prostate-specific membrane antigen (PSMA) immunoexpression in the neovasculature of colorectal carcinoma in Egyptian patients. Pathol.-Res. Pract..

[84] Cuda T.J., Riddell A.D., Liu C., Whitehall V.L., Borowsky J., Wyld D.K., Burge M.E., Ahern E., Griffin A., Lyons N.J. (2020). PET imaging quantifying 68Ga-PSMA-11 uptake in metastatic colorectal cancer. J. Nucl. Med..

[85] Hofman M.S., Emmett L., Violet J.Y., Zhang A., Lawrence N.J., Stockler M., Francis R.J., Iravani A., Williams S., Azad A. (2019). TheraP: A randomized phase 2 trial of 177Lu-PSMA-617 theranostic treatment vs cabazitaxel in progressive metastatic castration-resistant prostate cancer (Clinical Trial Protocol ANZUP 1603). BJU Int..

[86] Zeng C., Ke Z.F., Yang Z., Wang Z., Yang S.C., Luo C.Q., Wang L.T. (2012). Prostate-specific membrane antigen: A new potential prognostic marker of osteosarcoma. Med. Oncol..

[87] Sasikumar A., Joy A., Pillai M.R., Alex T.M., Narayanan G. (2017). 68Ga-PSMA PET/CT in osteosarcoma in fibrous dysplasia. Clin. Nucl. Med..

[88] Heitkötter B., Trautmann M., Grünewald I., Bögemann M., Rahbar K., Gevensleben H., Wardelmann E., Hartmann W., Steinestel K., Huss S. (2017). Expression of PSMA in tumor neovasculature of high grade sarcomas including synovial sarcoma, rhabdomyosarcoma, undifferentiated sarcoma and MPNST. Oncotarget.

[89] Vaz S., Oliveira C., Castanheira J.C., Silva Â.F., Costa D.C. (2018). Gastric GIST incidentally detected on 68Ga-PSMA-PET/CT: Correlation between functional imaging and histology. Clin. Nucl. Med..

[90] Sirtl S., Todica A., Ilhan H., Zorniak M., Bartenstein P., Mayerle J. (2021). Incidental finding of a PSMA-positive pancreatic cancer in a patient suffering from a metastasized PSMA-positive prostate cancer. Diagnostics.

[91] Lu Y., Li C. (2022). Incidental findings of coexisting metastatic pancreatic cancer in patients with prostate cancer on 18F–Prostate-specific membrane antigen PET/CT. Clin. Nucl. Med..

[92] Chan M., Schembri G.P., Hsiao E. (2017). Serous cystadenoma of the pancreas showing uptake on 68Ga PSMA PET/CT. Clin. Nucl. Med..

[93] Mhawech-Fauceglia P., Zhang S., Terracciano L., Sauter G., Chadhuri A., Herrmann F.R., Penetrante R. (2007). Prostate-specific membrane antigen (PSMA) protein expression in normal and neoplastic tissues and its sensitivity and specificity in prostate adenocarcinoma: An immunohistochemical study using mutiple tumour tissue microarray technique. Histopathology.

[94] Ren H., Zhang H., Wang X., Liu J., Yuan Z., Hao J. (2014). Prostate-specific membrane antigen as a marker of pancreatic cancer cells. Med. Oncol..

[95] Stock K., Steinestel K., Wiesch R., Mikesch J.H., Hansmeier A., Trautmann M., Beller N., Rehkämper J., Wardelmann E., Heitkötter B. (2017). Neovascular prostate-specific membrane antigen expression is associated with improved overall survival under palliative chemotherapy in patients with pancreatic ductal adenocarcinoma. BioMed Res. Int..

[96] Krishnaraju V.S., Kumar R., Mittal B.R., Sharma V., Singh H., Nada R., Bal A., Rohilla M., Singh H., Rana S.S. (2021). Differentiating benign and malignant pancreatic masses: Ga-68 PSMA PET/CT as a new diagnostic avenue. Eur. Radiol..

[97] Gala J.L., Loric S., Guiot Y., Denmeade S.R., Gady A., Brasseur F., Heusterspreute M., Eschwege P., Nayer P.D., Van Cangh P. (2000). Expression of prostate-specific membrane antigen in transitional cell carcinoma of the bladder: Prognostic value?. Clin. Cancer Res..

[98] Samplaski M.K., Heston W., Elson P., Magi-Galluzzi C., Hansel D.E. (2011). Folate hydrolase (prostate-specific antigen) 1 expression in bladder cancer subtypes and associated tumor neovasculature. Mod. Pathol..

[99] Gupta M., Choudhury P.S., Gupta G., Gandhi J. (2016). Metastasis in urothelial carcinoma mimicking prostate cancer metastasis in Ga-68 prostate-specific membrane antigen positron emission tomography-computed tomography in a case of synchronous malignancy. Indian J. Nucl..

[100] Campbell S.P., Baras A.S., Ball M.W., Kates M., Hahn N.M., Bivalacqua T.J., Johnson M.H., Pomper M.G., Allaf M.E., Rowe S.P. (2018). Low levels of PSMA expression limit the utility of 18F-DCFPyL PET/CT for imaging urothelial carcinoma. Ann. Nucl. Med..

[101] Wernicke A.G., Kim S., Liu H., Bander N.H., Pirog E.C. (2017). Prostate-specific membrane antigen (PSMA) expression in the neovasculature of gynecologic malignancies: Implications for PSMA-targeted therapy. Appl. Immunohistochem. Mol. Morphol..

[102] Hofstetter G., Grech C., Pils D., Pammer J., Neudert B., Pötsch N., Baltzer P., Traub-Weidinger T., Seebacher V., Aust S. (2022). Prostate-Specific Membrane Antigen (PSMA) Expression in Tumor-Associated Neovasculature Is an Independent Prognostic Marker in Patients with Ovarian Cancer. J. Pers. Med..

[103] Aide N., Poulain L., Elie N., Briand M., Giffard F., Blanc-Fournier C., Joly F., Lasnon C. (2021). A PSMA-targeted theranostic approach is unlikely to be efficient in serous ovarian cancers. EJNMMI Res..

[104] Chang S.S., Reuter V.E., Heston W.D., Bander N.H., Grauer L.S., Gaudin P.B. (1999). Five different anti-prostate-specific membrane antigen (PSMA) antibodies confirm PSMA expression in tumor-associated neovasculature. Cancer Res..

[105] Haffner M.C., Laimer J., Chaux A., Schäfer G., Obrist P., Brunner A., Kronberger I.E., Laimer K., Gurel B., Koller J.B. (2012). High expression of prostate-specific membrane antigen in the tumor-associated neo-vasculature is associated with worse prognosis in squamous cell carcinoma of the oral cavity. Mod. Pathol..

[106] Snow H., Hazell S., Francis N., Mohammed K., O’Neill S., Davies E., Mansfield D., Messiou C., Hujairi N., Nicol D. (2020). Prostate-specific membrane antigen expression in melanoma metastases. J. Cutan. Pathol..

[107] Brada M.D., Rushing E.J., Bächinger D., Zoller L., Burger I.A., Hüllner M.W., Moch H., Huber A., Eckhard A.H., Rupp N.J. (2022). Immunohistochemical expression pattern of theragnostic targets SSTR2 and PSMA in endolymphatic sac tumors: A single institution case series. Head Neck Pathol..

[108] Simsek D.H., Civan C., Ekenel M., Kuyumcu S., Sanli Y. (2021). 177Lu-PSMA therapy for metastatic testicular mixed germ cell tumor. Clin. Nucl. Med..

[109] Milowsky M.I., Nanus D.M., Kostakoglu L., Sheehan C.E., Vallabhajosula S., Goldsmith S.J., Ross J.S., Bander N.H. (2007). Vascular targeted therapy with anti–prostate-specific membrane antigen monoclonal antibody J591 in advanced solid tumors. J. Clin. Oncol..

[110] Abramova O.B., Kaplan M.A., Grin M.A., Yuzhakov V.V., Suvorov N.V., Mironov A.F., Drozhzhina V.V., Churikova T.P., Kozlovtseva E.A., Bandurko L.N. (2021). Photodynamic therapy of melanoma b16 with chlorin E6 conjugated with a PSMA-ligand. Bull. Exp. Biol. Med..

[111] Civan C., Isik E.G., Simsek D.H. (2021). Metastatic poorly differentiated thyroid cancer with heterogeneous distribution of 18F-FDG, 68Ga-DOTATATE, and 68Ga-PSMA on PET/CT. Clin. Nucl. Med..

[112] Lengana T., Lawal I.O., Mokoala K., Vorster M., Sathekge M.M. (2019). 68Ga-PSMA: A one-stop shop in radioactive iodine refractory thyroid cancer?. Nucl. Med. Mol. Imaging.

[113] Lütje S., Gomez B., Cohnen J., Umutlu L., Gotthardt M., Poeppel T.D., Bockisch A., Rosenbaum-Krumme S. (2017). Imaging of prostate-specific membrane antigen expression in metastatic differentiated thyroid cancer using 68Ga-HBED-CC-PSMA PET/CT. Clin. Nucl. Med..

[114] Santhanam P., Russell J., Rooper L.M., Ladenson P.W., Pomper M.G., Rowe S.P. (2020). The prostate-specific membrane antigen (PSMA)-targeted radiotracer 18F-DCFPyL detects tumor neovasculature in metastatic, advanced, radioiodine-refractory, differentiated thyroid cancer. Med. Oncol..

[115] Taywade S.K., Damle N.A., Bal C. (2016). PSMA Expression in papillary thyroid carcinoma: Opening a new horizon in management of thyroid cancer?. Clin. Nucl. Med.

[116] Sasikumar A., Joy A., Pillai M.R.A., Oommen K.E., Jayakumar R. (2018). Rare case of intratracheal metastasis detected on 68Ga-Prostate-specific membrane antigen PET/CT scan in a case of thyroglobulin elevated negative iodine scan syndrome. Clin. Nucl. Med..

[117] Zhao B., Dong A., Zuo C. (2022). Prostate-specific membrane antigen-avid bone metastases from urothelial carcinoma of the bladder. Clin. Nucl. Med..

[118] Parihar A.S., Sood A., Mittal B.R., Kumar R., Singh H., Dhatt S.S. (2020). 68Ga-PSMA-HBED-CC PET/CT and 18F-FDG PET/CT in ewing sarcoma. Clin. Nucl. Med..

[119] Can C., Gündoğan C., Kömek H. (2021). Is 68Ga-Prostate-specific membrane antigen PET/CT superior than 18F-FDG PET/CT for evaluation of metastatic osteosarcoma?. Clin. Nucl. Med..

